# Research and Application for Grey Relational Analysis in Multigranularity Based on Normality Grey Number

**DOI:** 10.1155/2014/312645

**Published:** 2014-02-16

**Authors:** Jin Dai, Xin Liu, Feng Hu

**Affiliations:** ^1^College of Software Engineering, Chongqing University of Posts and Telecommunications, Chongqing 400065, China; ^2^College of Computer Science, Chongqing University of Posts and Telecommunications, Chongqing 400065, China

## Abstract

Grey theory is an essential uncertain knowledge acquisition method for small sample, poor information. The classic grey theory does not adequately take into account the distribution of data set and lacks the effective methods to analyze and mine big sample in multigranularity. In view of the universality of the normal distribution, the normality grey number is proposed. Then, the corresponding definition and calculation method of the relational degree between the normality grey numbers are constructed. On this basis, the grey relational analytical method in multigranularity is put forward to realize the automatic clustering in the specified granularity without any experience knowledge. Finally, experiments fully prove that it is an effective knowledge acquisition method for big data or multigranularity sample.

## 1. Introduction

Grey theory [1] is an effective uncertainty knowledge acquisition model, proposed by Professor Deng. It mainly focuses on small sample and limited information, which is only known partially.

Grey relational analysis [2] is an important task of grey theory by which to scale the similar or different level of development trends among various factors. It has drawn more and more researchers' attention in recent years and achieved many research results. In paper [3], grey relational degree of decision-making information was defined to solve the decision-makers clustered situation. A decision method based on grey relational analysis and D-S evidence theory was proposed to reduce the uncertainty of decision significantly [4]. In paper [5], the convexity of data was used to characterize the similarity of the samples, and the concept of 3D grey convex relation degree was put forward. The MYCIN uncertain factor in fuzzy set theory and grey relation method were combined to build an inferential decision model [6]. In paper [7], the grey degree was seen as the classification standard of objects' uncertainty. Then, a novel grey degree and grey number grading method was proposed based on set theory. The authors gave the decision method of quantitative and qualitative change in a scheme and the measure index of qualitative change judged by evaluator, and then, time weighted ensuring model was constructed based on grey relational degree [8]. The computing method of relational degree was defined based on information reduction operator of interval grey numbers sequence, and multicriteria interval grey numbers relational decision-making model was constructed [9].

From the above research achievements, it should be clear that the researchers have paid the more and more attention on the distribution of data in order to reduce the uncertainty with the development of grey relational analysis field. Simultaneously, other uncertainty knowledge acquisition methods are combined with it to supply a better application. However, we do not have the research on multigranularity grey relational analysis for data sequence, especially large data.

How to build the information granules with a strong data presentation and efficient processing capabilities is the most important for multigranularity relational analysis. In this paper, combining with the probability distribution of the data, the conception of the normality grey number is proposed. Moreover, the corresponding grey degree and grey relational degree are given. Finally, the method of grey relational analysis in multigranularity is constructed. Without any prior knowledge, it enables automatic clustering in the specified granularity. The experiments show the effectiveness of this method which provides a novel thought based on grey theory for big data knowledge acquisition.

## 2. Grey Theory Based on Normal Distribution

### 2.1. Grey Number and Grey Degree

**Definition 1 (grey number [1])**

A grey number is an uncertain number in an interval or a data set, denoted as “⊗”. That is, $⊗\in [\underline{a},\overset{-}{a}]$, where $\underline{a}$ and $\overset{-}{a}$ are, respectively, the infimum and supremum. When $\underline{a}$ and $\overset{-}{a}\to \infty$, ⊗ is called black number, else when $\underline{a}=\overset{-}{a}$, ⊗ is called white number.

Grey number can be whitening with our understanding towards them. Usually, the whitenization weight function [1] should be used to describe the varying preferences of a grey number in the value range. Grey whitenization weight function presents the subjective information about the grey number, and the grey degree of a grey number is the level, which presents the amount of information. The grey degree of grey number of a typical whitenization weight function was given by Professor Deng:

(1) $$\begin{array}{c} g^{{}^{\circ}}\left(⊗\right)=\frac{2\left|b_{1}-b_{2}\right|}{b_{1}+b_{2}}+\max\left\{\frac{\left|a_{1}-b_{1}\right|}{b_{1}},\frac{\left|a_{2}-b_{2}\right|}{b_{2}}\right\}. \end{array}$$

Based on the length of grey interval *l*(⊗) and the mean whitening number of a grey number $\hat{⊗}$, an axiomatic definition [10] about grey degree was given by Professor Liu et al.: $g^{{}^{\circ}}(⊗)=l(⊗)/\hat{⊗}$. However, there is a problem in preceding definitions about grey degree. When *l*(⊗) → *∞*, the grey degree may tend to infinity. It is not normative. Consequently, a definition of grey degree based on the emergence background and domain was proposed by Professor Liu et al.: *g*°(⊗) = *μ*(⊗)/*μ*(*Ω*), where *μ*(⊗) is the measure of grey number field and *μ*(*Ω*) is the measure of domain [10]. Entropy grey degree from Professor Zhang and Qin is: *g*° = *H*/*H*_*m*_, where *H* and *H*_*m*_ expresses the entropy and maximum entropy of grey number [11]. This definition requires that the values of grey number in the interval are discrete; it is not appropriate for continuous values.

### 2.2. Normal Distribution Grey Number and Its Grey Degree

A phenomenon usually is similar to a normal distribution when it is decided by the sum of several independent and slight random factors and the effect of every factor is respectively and evenly small. The normal distribution is widely existed in natural phenomenon, society phenomenon, science and technology and production activity. Much random phenomenon in practice obeys or similarly obeys normal distribution.

**Definition 2 (normality grey number)**

It is an uncertain number in the interval [*a*, *b*] or the data set, where *a* and *b* are the infimum and supremum. The value of this number obeys the normal distribution where the mean is *μ* and the deviation is *σ* in [*a*, *b*]. The normality grey number is denoted as ⊗_*N*(*μ*,*σ*^2^)_, abbreviated as ⊗_(*μ*,*σ*^2^)_.

**Figure 1:**
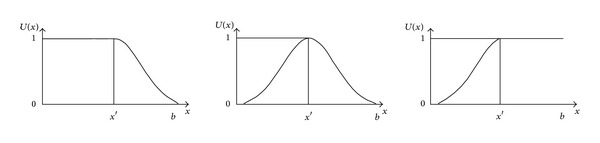
The typical whitenization weight function of normality grey number.

On the basis of the definition of normality grey number, its typical whitenization weight function could be given (Figure 1).

The definition of grey degree based on normal distribution has an universal significance. According to Lindeberg-Levy central limit theorem, any of the random variables generated by probability distributions, when it is under operation of sequence summing or equivalent arithmetic mean, would be unified induced to normal distribution; that is,

(2) $$\begin{array}{c} \frac{\sqrt{n}\left(\overset{-}{X}-\mu \right)}{\sigma }⟶N\left(0,1\right),\;n⟶\infty . \end{array}$$

According to the property of normal distribution, the normality grey number has the following properties.

Given ⊗_(*μ*_*i*_,*σ*_*i*_^2^)_, *a*, *b* is real number, then *a* ⊗_(*μ*_*i*_,*σ*_*i*_^2^)_ + *b* = ⊗_(*aμ*_*i*_+*b*,*a*^2^*σ*_*i*_^2^)_.

Given ⊗_(*μ*_*i*_,*σ*_*i*_^2^)_ and ⊗_(*μ*_*j*_,*σ*_*j*_^2^)_, then ⊗_(*μ*_*i*_,*σ*_*i*_^2^)_ ± ⊗_(*μ*_*j*_,*σ*_*j*_^2^)_ = ⊗_(*μ*_*i*_±*μ*_*j*_,*σ*_*i*_^2^+*σ*_*j*_^2^)_.

Expectation *μ* and deviation *σ* of normal distribution are used to denote the distribution of continuous random variable. There is a higher probability when it gets closer to *μ*, conversely, the probability will be lower. Deviation *σ* embodies the concentration of variable distribution. The more concentrated for normal distribution, the larger the deviation. In normal distribution, the value of random variable is a striking feature, that is “3*σ*” principle.

**Figure 2:**
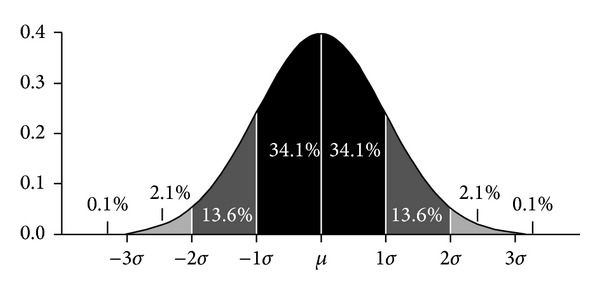
Value distribution of normal distribution.

As shown in Figure 2, the value distribution in [*μ* − 3*σ*, *μ* + 3*σ*] has achieved 99%, and this interval plays a crucial role in the whole distribution. So, the paper proposes the definition of grey degree based on normal distribution.

**Definition 3 (grey degree based on normal distribution)**

Given normality grey number ⊗_*N*(*μ*,*σ*^2^)_ ∈ *Ω*, then *g*°(⊗) ≈ 6*σ*/*μ*(*Ω*), *μ*(*Ω*) is the domain measure.

According to Definition 3, the formal description of grey number based on normal distribution is ${\hat{⊗}}_{\left(g^{{}^{\circ}}\right)}\approx \mu_{\left(6\sigma /|b-a|\right)}$, where $\hat{⊗}$ is the kernel of grey number which is usually the expectation *μ* of grey number in domain, and |*b* − *a*| is the grey number which is usually the upper bound or lower bound.

Grey number based on normal distribution provides a novel thought based on grey system for multigranularity knowledge acquisition under large data set.

### 2.3. Hypothesis Testing and Transformation of Normal Distribution

Although normal distribution has favorable universality, many data distributions do not conform to normality assumption, such as *F* distribution, Γ distribution, and *χ* ^2^ distribution. So, whether a given data set can transform into a grey number based on normal distribution needs the hypothesis testing of normal distribution. There are many methods for the whole normal testing, such as Jarque-Bera [12] testing method (be applied to large scale sample) and Lilliefors [13] normal testing method (be generally used).

If the data set does not satisfy normal testing, it can be transformed into normal distribution through some methods, such as power transformation of Box and Cox [14], distribution curve of Johnson [15], item group or packing [16], and Bootstrap resampling [17]. It should be noted that the data transformation is not entirely accurate. If the data set is not normal distribution itself, there will be a new error after normal transformation.

## 3. Grey Relational Analysis Based on Normality Grey Number

### 3.1. Grey Relational Analysis

Grey relational analysis is a method for quantitatively describing and comparing to the development tendency of a system. The core idea is to compare the geometrical similarity between reference data sequence and several comparative data sequences. The higher the grey correlation, the closer these sequences about their development direction and rate, and their relationship will be closer to each other.

**Definition 4 (grey absolutely relational degree)**

Let  *X*_*i*_ and *X*_*j*_ be both the equally spaced sequence [10], and let *X*_*i*_^0^ = (*x*_*i*_^0^(1), *x*_*i*_^0^(2),…, *x*_*i*_^0^(*n*)), *X*_*j*_^0^ = (*x*_*j*_^0^(1), *x*_*j*_^0^(2),…, *x*_*j*_^0^(*n*)), respectively, be the zeroed value of start point [10]. Then, the grey absolutely relational degree of *X*_*i*_ and *X*_*j*_ is

(4) $$\begin{array}{c} \varepsilon_{ij}=\frac{1+\left|s_{i}\right|+\left|s_{j}\right|}{1+\left|s_{i}\right|+\left|s_{j}\right|+\left|s_{i}-s_{j}\right|},\\ \left|s_{i}-s_{j}\right|=\left|\sum_{k=2}^{n-1}\left(x_{i}^{0}\left(k\right)-x_{j}^{0}\left(k\right)\right)+\frac{1}{2}\left(x_{i}^{0}\left(n\right)-x_{j}^{0}\left(n\right)\right)\right|,\\ \left|s\right|=\left|\sum_{k=2}^{n-1}x^{0}\left(k\right)+\frac{1}{2}x^{0}\left(n\right)\right|. \end{array}$$

In addition, there is relatively relational degree in grey relational analysis. Its construction is similar to absolutely relational degree. A little difference is that *X*_*i*_ and *X*_*j*_ will be processed by initial value before computing the zeroed value of start point.

### 3.2. Grey Relational Degree Based on Normal Grey Number

Grey absolutely relational degree is the basic of grey relational analysis. However, carefully analyzing Definition 4, there are the following problems about grey absolutely relational degree.

(1) The length of sequence *X*_*i*_ and *X*_*j*_ must be equal; otherwise we need to fill the missing data or delete the excess data of the longer sequence which increases the uncertainty of system and has a direct impact on values of relational degree. (2) Grey absolutely relational degree in fact is for white number. The relational degree will not work if the sequence itself is uncertain or the element of sequence is grey number directly.

**Figure 3:**
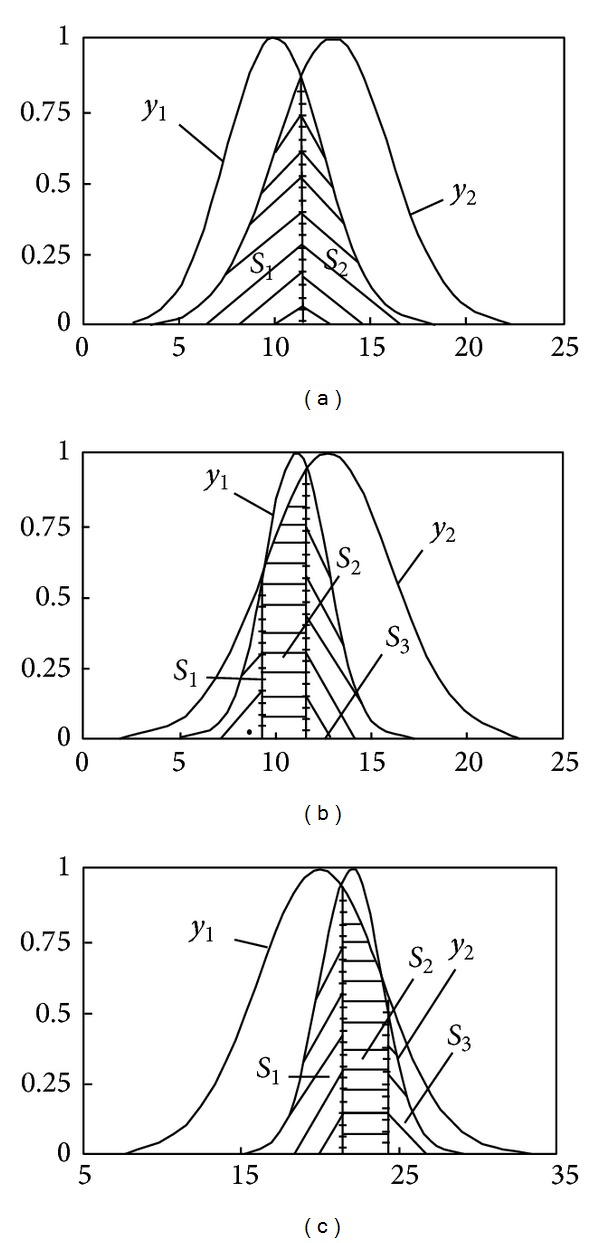
Similar situation between normal distributions.

Based on above analysis, combining with the property of normality grey number, a grey relational degree based on normality grey number is proposed. Normal random distribution is the core of this relational degree, which is obtained by calculating the area of two intersecting normal random distributions. The intersecting stations of two normal distributions are shown in Figure 3 (the shadow area presents each other's similarity).

Set *X*_1_ ~ *N*(*μ*_1_, *σ*_1_), *X*_2_ ~ *N*(*μ*_2_, *σ*_2_), *y*_1_(*x*) and *y*_2_(*x*) as, respectively, the distribution function of *X*_1_ and *X*_2_, where *x*_0_ is the intersection of the two curves *y*_1_ and *y*_2_. Then, the intersecting area of *X*_1_ and *X*_2_ is

(4) S=∫−∞x0y2(x)dx+∫x0∞y1(x)dx=∫−∞z2ϕ(z)dz+∫z1∞ϕ(z)dz,

where *z*_1_ = (*x*_0_ − *μ*_1_)/*σ*_1_, *z*_2_ = (*x*_0_ − *μ*_2_)/*σ*_2_, *ϕ*(*z*) is standard normal distribution. If *x*_0_ is known, *z*_1_ and *z*_2_ are then obtained. Enquiring the table of standard normal distribution, the intersecting area *S* can be calculated.

According to the intersection between distribution curves |*z*_1_ | = |*z*_2_|, then obtain that

(5) $$\begin{array}{c} x_{0}^{\left(1\right)}=\frac{\mu_{2}\sigma_{1}-\mu_{1}\sigma_{2}}{\sigma_{1}-\sigma_{2}},\;\;x_{0}^{\left(2\right)}=\frac{\mu_{1}\sigma_{2}+\mu_{2}\sigma_{1}}{\sigma_{1}+\sigma_{2}}. \end{array}$$

In light of “3*σ*” principle of normal distribution, 99.74% of values are in [*μ* − 3*σ*, *μ* + 3*σ*]. So, when calculating the similarity of two normal distributions, we only consider the distribution of variables in the interval. It would be well as if set *μ*_1_ ≤ *μ*_2_, then, there are the following three situations about the distribution of *x*_0_^(1)^ and *x*_0_^(2)^:

(1) If *x*_0_^(1)^,  *x*_0_^(1)^ ∉ [*μ*_2_ − 3*σ*_2_, *μ*_1_ + 3*σ*_1_], indicating that the values distribution of intersections can be neglected, so *S* = 0.
(2) If there is a point *x*_0_^(1)^ or *x*_0_^(2)^ in the interval [*μ*_2_ − 3*σ*_2_, *μ*_1_ + 3*σ*_1_], as shown in Figure 3(a), then
  (6) S=S1+S2=∫−∞z2ϕ(x)dx+∫z2∞ϕ(x)dx=∫−∞z2ϕ(x)dx+(1−∫−∞z1ϕ(x)dx),
  where *z*_1_ = (*x*_0_ − *μ*_1_)/*σ*_1_,  *z*_2_ = (*x*_0_ − *μ*_2_)/*σ*_2_.
(3) If *x*_0_^(1)^ and *x*_0_^(2)^ are in the interval [*μ*_2_ − 3*σ*_2_, *μ*_1_ + 3*σ*_1_] simultaneously, as shown in Figures 3(b) and 3(c), then,
  (7) S=S1+S2+S3={∫−∞z  1(1)ϕ(x)dx+(∫−∞z2(2)ϕ(x)dx−∫−∞z2(1)ϕ(x)dx)+∫z1(2)∞ϕ(x)dx σ1>σ2∫−∞z2(1)ϕ(x)dx+(∫−∞z1(2)ϕ(x)dx−∫−∞z1(1)ϕ(x)dx)+∫z2(2)∞ϕ(x)dx σ1≤σ2,
  where *x*_0_^(1)^ ≤ *x*_0_^(2)^,  *z*_*i*_^(*j*)^ = (*x*_0_^(*j*)^ − *μ*_*i*_)/*σ*_*i*_.

Considering the normalization of the similarity, *S* must be normalized. The area is seen as the similarity of two normal distributions after it is normalized.

**Definition 5 (similarity of normal grey number)**

Given normality grey number ⊗_(*μ*_1_,*σ*_1_)_, ⊗_(*μ*_2_,*σ*_2_)_, their relational degree is:

(8) $$\begin{array}{c} \gamma \left({⊗}_{\left(\mu_{1},\sigma_{1}\right)},{⊗}_{\left(\mu_{2},\sigma_{2}\right)}\right)=\frac{2S}{\left(\sqrt{2\pi }\left(\sigma_{1}+\sigma_{2}\right)\right)}, \end{array}$$

where *S* is the intersection area between ⊗_(*μ*_1_,*σ*_1_)_ and ⊗_(*μ*_2_,*σ*_2_)_. Similarity of normal grey number is abbreviated as normality grey relational degree.

Similarity of normal grey number better considers the change of data in interval to show the property of its probability distributions and to be fundamental for grey relational analysis based on normality grey number.

### 3.3. Unsupervised Fast Grey Clustering Based on Grey Relational Degree

Grey clustering is a method to divide some observation index or object into several undefined categories according to the grey number relational matrix or whiten weight function of grey number. The basic ideology is to nondimensionalize the evaluating indicator of original observation data, to calculate the relation coefficient, relational degree and to sort the evaluation indicator according to relational degree. The grey clustering only supports small sample data at present, and the whole process needs manual intervention. So, it will increase complexity and uncertainty of the algorithm. Because of this analysis, unsupervised fast grey clustering based on grey relational degree is proposed in this paper. With core of grey relational degree and by constructing grey relational matrix, this algorithm realizes unsupervised dynamical clustering using improved *k*-means method.

The analysis of the improved algorithm is as follows. Features that are most similar will divide into the same category while sample data is classified. Their relationship in space can be characterized by some norm measure. Through gradual improvements in their layers (i.e., clustering number), the relationship of categories between layers is changing with the increase of layers. This change can be portrayed by some rules. The clustering will finish, once the requirements of the rules are met. In this paper, standard deviation of samples *S*_*i*_ is used to characterize this change. With the increase of clustering layers, the various categories will get more and more aggregative and *S*_*i*_ will continue to decrease. Judging if the clustering has finished, the mean sample variance ${\overset{-}{S}}_{n}$ is used as convergence conditions when *K* = *n* (*n* is the number of samples); that is, clustering will be accomplished when $\min(S_{i})<{\overset{-}{S}}_{n},(i=1,\ldots ,K)$.

Given grey relational matrix *R*:

(9) $$\begin{array}{c} R=\left[\begin{array}{cccc} \gamma_{11} & \gamma_{12} & \cdots & \gamma_{1m}\\ & \gamma_{22} & & \gamma_{2m}\\ & & \ddots & \vdots \\ & & & \gamma_{mm} \end{array}\right], \end{array}$$

where *γ*_*ij*_ is the grey relational degree of grey number ⊗_*i*_ and ⊗_*j*_, it could be grey number which is irregular data or satisfy one distribution, for example, normal distribution. According to grey relational matrix, unsupervised fast grey clustering based on grey relational degree is proposed.

**Algorithm 6 (unsupervised fast grey clustering based on grey relational degree)**

 Input: given *n* sequences, the length of them is *m*
  (10) X1=(x1(1),x1(2),…,x1(m)),X2=(x2(1),x2(2),…,x2(m)),⋮Xn=(xn(1),xn(2),…,xn(m)).
 Output: aggregation matrix *R*′ = {*X*_*i*_, *Groupid*}, *i* = 1,2,…, *n*; *Groupid* is category number.

*Steps*.

1. Calculating grey relational matrix: *R* = {*X*_*i*_, *X*_*j*_, *sim*, *Clusterid*}//*sim* is grey relational degree of the sample *X*_*i*_, *X*_*j*_ (*i* < *j*), *Group* is clustering number, whose invital value is equal to zero.
2. Extracting all unrepeated *sim* in *R* to construct an ascending category vector *C* = {*sim*, *Clusterid*}.
3. Calculating threshold $e,e={\overset{-}{S}}_{n}$//the control condition of clustering finished.
4. Initial category *K* = 1, *v* = 0//*v* is control variable of circulating.
5. *Do*
  1. Constructing center category table *TC*: divide *C* into *K* + 1 portions equally, and take former *K* portions to join *TC* as the initial category of *C* under the case of *K*; set *Groupid* = 0.
  2. Set *e*_1_ = 0//*e*_1_ as temporary control variable.
  3. While *e*_1_ ≠ *v*, execute the following circulating://after clustering tends to stabilize, the standard deviation of all categories will converge to a steady value.
    1. *e*_1_ = *v*,
    2. calculating the distance of every value in *C*′ and all categories in *TC*, and merging it into the category where distance is minimum,
    3. revising the center distance of all categories in *TC* according to weighted average,
    4. computing the standard deviation *S*_*i*_ of all categories in *T*, set *v* = min⁡(*S*_*i*_).
  4. *K* = *K* + 1. While (*v* > *e*)//when *v* ≤ *e*, the aggregation of all categories will be ok, and clustering finishes.
6. Update *Groupid in R* by the corresponding *sim* in *C*.
7. Set *Groupid* = 1, *X*_*i*_ in ascending order and *Clusterid* in descending order, *R* will be processed as follows:
  1. Taking *x*_*k*_ ∈ *X*_*i*_ and *x*_*k*_ ∉ *R*′ in turn, *c*_*i*max⁡_ is the largest category number in *X*_*i*_. Then, the most similar sample set of *x*_*k*_ in *R* is
    1. *R*′ = {(*x*_*k*_, *Groupid*)}∪{(*x*_*m*_, *Groupid*) | *x*_*m*_ ∈ *X*_*j*_,
    2. *X*_*i*_ = *x*_*k*_∧*Clusterid* = *c*_*i*max⁡_}//the bigger *Clusterid* is, the more similar between clusters.
  2. *R*′ = *R*′ ∪ {(*x*_*k*_, *c*_*i*max⁡_)}//the most similar sample of every cluster.
  3. *Groupid* = *Groupid* + 1.
8. Return *R*′.

After clustering by Algorithm 6, we can obtain the equivalent cluster of samples under grey relational degree. The same *Groupid* is similar sample sequence.

Using classic grey relational degree as the calculating basic of similar sequence, Algorithm 6 has strict requirement to the length of sequence and sequence values. However, the comparative similar sequence is sequence distribution under normality grey relational degree; there is not a rigid requirement for the length of sequences and it is suitable for grey relational analysis of large and multigranularity samples. For example, given the power consumption of one city in a year (by the day), we need to analyze and make statistics of the power consumption of a week, a month, and a quarter. The traditional grey relational analysis method cannot do this.

### 3.4. Multigranularity Grey Clustering Algorithm Based on Normal Grey Domain

Granularity is a concept from physics which is mean measure for the size of microparticle and the thickness of information in artificial intelligence field. Physics granularity involves the refinement partition to physics objects, but granularity information is to measure information and knowledge refinement [18, 19]. The essence of granular computing is to select appropriate granularity and to reduce complexity of solution problem.

Let *R* denote the set which is composed of all equivalence relation on *X*, and the equivalence relation can be defined as follows.

**Definition 7 (the thickness of granularity)**

Set *R*_1_, *R*_2_ ∈ *R*, if ∀*x*, *y* ∈ *X*, *xR*_1_ *y*⇒*xR*_2_ *y*, then it is called that *R*_1_ is finer than *R*_2_, denoted as *R*_1_ ≤ *R*_2_.

Definition 7 expresses the “coarse” and “fine” concept of granularity. So, the following theorems can be proved [4].

**Theorem 8**

According to the above definition about the relationship “≤”, *R* forms a comprehensive semisequence lattices.

Theorem 8 is very revealing about the core property of granularity. Based on the theorem, the following sequence would be obtained:

(11) $$\begin{array}{c} R_{n}\leq R_{n-1}\cdots \leq R_{1}\leq R_{0}. \end{array}$$

The above sequence is visually correspondent to an *n*-level tree. Set *T* as an *n*-level tree, all its leaf nodes construct a set *X*. Then the nodes in every level correspond to a partition of *X*. The hierarchical diagram of clustering obtaining by clustering operating also is an *n*-level tree. Therefore, there must be a corresponding sequence of equivalence relation. So, this fully shows that there is good communicating peculiarity between clustering and granularity. It is the theory basic of the proposed algorithm.

According to the property of granular computing and Algorithm 6, multigranularity grey clustering algorithm based on normality grey domain is proposed. The algorithm does a partition under the designated granularity based on time sequence then dynamic clustering. The algorithm includes data partitioning of sequence, normality grey sequence constructing, and grey clustering. Take the data of time sequence, for example, to elaborate the algorithm as follows.

Given time sequence *X* ^(0)^ = (*x* ^(0)^(1), *x* ^(0)^(2),…, *x* ^(0)^(*n*)) and the necessary granularity *L* for analysis, there is the following partition for original time sequence:

(12) X(0)=(x(0)(1),x(0)(2),…,x(0)(L)︸L,…,x(0)(mL+1),x(0)(mL+2),…,x(0)(mL+L))︸L(m=1,…,nL).

It should be noted that at the designated granularity partition of time sequence is incomplete. Number of the sequences is not necessarily exactly equal and values of subsequence may be in granularity *L*. The partitioning sequence is composed by *n*/*L* subsequences. Calculating the expectation *μ* and variance *σ* of every subsequence, the sequence *X* ^(0)^ can be transformed as normality grey sequence:

(13) XN(0)=(⊗(μ1,σ1)(0)(1),⊗(μ2,σ2)(0)(2),…,⊗(μk,σk)(0)(k)),k=1,2,…,nL.

After designated granularity partitioning to sequence, grey clustering algorithm based on normality grey domain could be obtained.

**Algorithm 9 (grey clustering algorithm based on normality grey domain)**

 Input: time granularity *L*,  *n* sequences and the length of them is *m*:
  (14) X1=(x1(1),x1(2),…,x1(m)),X2=(x2(1),x2(2),…,x2(m)),⋮Xn=(xn(1),xn(2),…,xn(m)).
 Output: aggregation matrix *R*′ = {*X*_*i*_, *Groupid*}, *i* = 1,2,…, *n*; *Groupid* is category number.

*Steps*.

1. Sequence granularity portioning: invoking the formula of sequence partition for sequences to do a partition, its granularity is *L*. Obtaining the partitioned sequence *X*_*i*_′(*k*), (*i* = 1,2,…, *n*; *k* = 1,2,…*m*/*L*).
2. Normality hypothesis testing to sequences. If they do not satisfy normal distribution, then make a normal distribution transformation.
3. Transforming *X*_*i*_′(*k*) into normality grey sequence *X*_*G*_*i*__′(*k*) = {⊗_(*μ*_*i*_,*σ*_*i*_)_(*k*)}.
4. Invoking Algorithm 6 to dynamic cluster for sequence *X*_*i*_′(*k*), obtaining *R*′.
5. Return *R*′.

## 4. Experiment Results and Analysis

In order to evaluate the performance of Algorithm 9, two data sets of UCI database are selected for experiments (details shown in Table 1).

Dataset 1 records the power consumption of a family in 4 years (taking samples in 1-minute interval, from October 1, 2006, to November 1, 2010) in which there are 2,049,280 effectively recorded items. Dataset 2 gathers the vehicle traffic data of a highway junction in Los Angeles by sensor in 5-minute interval. There are 47,497 effectively recorded items from April 10, 2005, to October 1, 2005.

According to the feature of dataset, we need to analyze the power consumption of Dataset 1 to find out the peaks and troughs in electricity demand by the month and to analyze the traffic station of highway in Dataset 2 by the hour.

Using Algorithm 9 to treat the Dataset 1, the time sequences are transformed into normal grey sequences under the designated time granularity (by the month) firstly, as shown in Table 2.

Clustering normality grey sequences by years under the designated granularity (by the month), the following clustering results can be obtained (sort by expectation in descending order):

(15) X2007={(1,2,3,11,12),(4,5,6,9,10),(7,8)},X2008={(1,2,3,11),(4,6,10,12),(5,9),(7),(8)},X2009={(1,11,12),(2,3,10,12),(4,5,9),(6,8),(7)},X2010={(1,2),(3,5,10,11),(4,6,9),(7,8)}.

**Figure 4:**
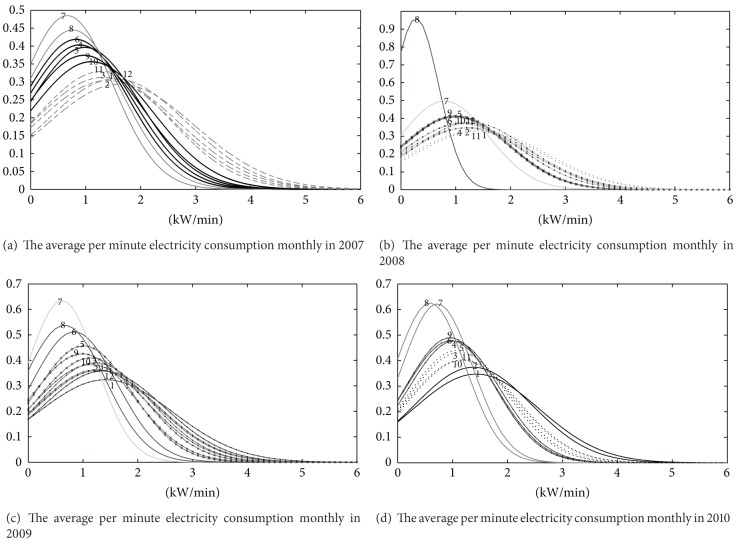
Cluster distribution of dataset 1.

We could see that the most power consumption happened in January, February, November, and December and increased steadily; the more power consumption is in March, April, May, June, September, October and the change is relative stability; the least consumption is in July and August, and the fluctuation is the most evident. The clustering result is shown in Figure 4.

Using Algorithm 9 to treat the Dataset 2, the time sequences are transformed into normality grey sequences under the designated time granularity (by the hour) firstly, as shown in Table 3.

Clustering normal grey sequences by years under designated granularity (by the hour), the following clustering results can be obtained (sort by expectation in descending order):

(16) X={(9,14,15,16,17),(10,11,12,13,19), (7,8),(20,21,22),(6,23),(0,5),(1,2),(3,4)}.

**Figure 5:**
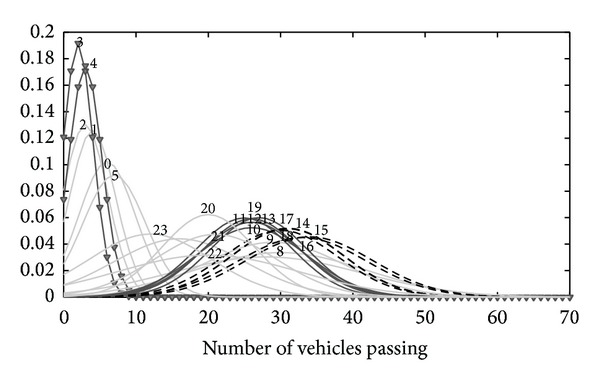
Cluster distribution of dataset 2.

The clustering results show that there is daily the most heavy traffic at 9, 14, 15, 16, and 17 o'clock and the volume is relative stability; the more heavy traffic is at 10, 11, 12, 13, and 19 o'clock, and the fluctuation of volume is also not very violent; the least busy hour is at 3, 4 o'clock in every morning and the volume is most low and most fluctuant. The clustering result is shown in Figure 5.

As shown in Figures 4 and 5, after multigranularity clustering using Algorithm 9, the results better match the actual data distribution. To further compare the clustering performance of Algorithm 9, the classic *k*-means and DBSCAN [20] clustering algorithm are introduced into this experiment. The datasets are all transformed as normality grey sequences for fairness of experiment. *k*-means and DBSCAN need to appoint the number of categories or set the optimum parameters artificially, but there is not any experience knowledge in Algorithm 9. The performance of three algorithms is all under the best experiment results. The comparative criteria contain entropy and purity [20]. The best algorithm has the lowest entropy and highest purity. The experiment results are in Table 4.

Table 4 shows that the performance of Algorithm 9 is obviously superior to DBSCAN and roughly equivalent to *k*-means. The clustering number of Algorithm 9 is almost the same as the other clustering algorithm, but the former is automatic clustering and does not need any experience knowledge.

## 5. Conclusion

Although grey theory is becoming mature, it is not widely applied in practice. An important reason is the lack of effective grey relational analysis method for large data or multigranularity sample. The concept of normality grey number is built and the corresponding grey degree is defined based on probability distributions. Meanwhile, multigranularity grey relational analysis method based on normality grey relational degree is proposed. The whole method can realize automatic clustering without the prior knowledge. Finally, the proposed method has a more effective and significant performance than other algorithms in the clustering experiments. In further research, we will focus on grey modeling and predicting models based on normality grey sequence and build a complete theory system about normality grey relational analysis and multigranularity simulation and prediction.

## Figures and Tables

**Table 1:** Test UCI datasets.

ID	Name of dataset	Number of items	Effective items	Attribute type
1	Individual household electric power consumption	2,075,259	2,049,280	Real
2	Dodgers loop sensor	50,400	47,497	Integer

**Table 2:** The converted normal grey sequence of dataset 1.

The average power consumption (kilowatt/minute)				
Month	2007	2008	2009	2010
1	(1.546033, 1.29201)	(1.459920, 1.2058)	(1.410202, 1.23113)	(1.430524, 1.14829)
2	(1.401083, 1.31234)	(1.181384, 1.15272)	(1.247567, 1.09718)	(1.375854, 1.06622)
3	(1.318627, 1.27604)	(1.245336, 1.14283)	(1.226734, 1.02127)	(1.130075, 0.922584)
4	(0.891188, 0.98919)	(1.115972, 1.07732)	(1.140689, 0.973492)	(1.027295, 0.836552)
5	(0.985861, 1.00641)	(1.024281, 0.964457)	(1.012855, 0.87253)	(1.095284, 0.904557)
6	(0.826814, 0.953)	(0.994096, 0.977501)	(0.840756, 0.779055)	(0.969614, 0.833514)
7	(0.667366, 0.822755)	(0.794780, 0.802558)	(0.618120, 0.628642)	(0.721067, 0.641291)
8	(0.764186, 0.896657)	(0.276488, 0.415126)	(0.664618, 0.742219)	(0.590778, 0.638138)
9	(0.969318, 1.06606)	(0.987680, 0.962621)	(0.986840, 0.936502)	(0.956442, 0.815049)
10	(1.103910, 1.11954)	(1.136768, 1.05956)	(1.144486, 1.04126)	(1.163398, 0.991783)
11	(1.294472, 1.21085)	(1.387065, 1.20713)	(1.274743, 1.10642)	(1.196854, 0.989863)
12	(1.626473, 1.3572)	(1.275189, 1.05568)	(1.364420, 1.11317)	

**Table 3:** The converted normal grey sequence of dataset 2.

Time	Expectations, variances
0	(6, 3.90564)
1	(4, 3.16916)
2	(3, 3.05947)
3	(2, 2.08197)
4	(3, 2.28398)
5	(7, 4.32885)
6	(16, 9.05729)
7	(28, 13.1354)
8	(30, 11.8488)
9	(29, 9.62101)
10	(26, 7.61425)
11	(25, 6.67066)
12	(26, 6.74319)
13	(27, 6.64526)
14	(31, 7.67199)
15	(34, 8.7623)
16	(33, 8.87094)
17	(31, 7.72262)
18	(30, 7.90683)
19	(26, 7.05241)
20	(20, 6.40364)
21	(21, 8.41713)
22	(20, 12.5605)
23	(12, 8.37453)

**Table 4:** Performance comparison among the algorithms.

Evaluation index	Dataset 1 (individual household electric power consumption)			Dataset 2 (Dodgers loop sensor)		
	*k*-means (*k* = 3)	DBSCAN (minpts = 1, eps = 0.01)	Algorithm 9	*k*-means (*k* = 7)	DBSCAN (minpts = 2, eps = 0.1)	Algorithm 9
Entropy	0.21	0.39	0.23	0.17	0.31	0.18
Purity	0.78	0.62	0.77	0.85	0.73	0.83
Clusters	3	4	4	7	8	8

## References

[1] Deng JL (2002). *The Foundation of Grey System*.

[2] Liu SF, Hu ML, Yang YJ (2012). Progress of grey system models. *Transactions of Nanjing University of Aeronautics and Astronautics*.

[3] Song J, Dang Y, Hua Z (2010). Study on group decision-making method based on grey cluster model. *Control and Decision*.

[4] Li P, Liu S (2011). Interval-valued intuitionistic fuzzy numbers decision-making method based on grey incidence analysis and D-S theory of evidence. *Acta Automatica Sinica*.

[5] Wu LF, Liu SF (2013). Panel data clustering method based on grey convex relation and its application. *Control and Decision*.

[6] Li P, Liu SF, Fang ZG (2012). Interval-valued intuitionistic fuzzy numbers decision-making method based on grey incidence analysis and MYCIN certainty factor. *Control and Decision*.

[7] Jian LR, Liu SF (2013). Definition of grey degree in set theory and construction of grey rough set models. *Control and Decision*.

[8] Wang HH, Zhu JJ, Fang ZG (2013). Aggregation of multi-stage linguistic evaluation information based on grey incidence degree. *Control and Decision*.

[9] Yang B, Fang Z, Zhou W, Liu J (2012). Incidence decision model of multi-attribute interval grey number based on information reduction operator. *Control and Decision*.

[10] Liu SF, Dang YG, Fang ZG (2010). *Grey System Theory and Its Applications*.

[11] Zhang QS, Qin H (1996). New definition of grey number's grade. *Journal of Northeast Petroleum University*.

[12] Thadewald T, Büning H (2007). Jarque-Bera test and its competitors for testing normality—a power comparison. *Journal of Applied Statistics*.

[13] Lilliefors HW (1967). On the Kolmogorov-Smirnov test for normality with mean and variance unknown. *Journal of the American Statistical Association*.

[14] Box GER, Cox DR (1964). An analysis of transformations. *Journal of the Royal Statistical Society B*.

[15] Farnum NR (1996). Using Johnson curves to describe non-normal process data. *Quality Engineering*.

[16] Hanson WE, Curry KT, Bandalos DL (2002). Reliability generalization of working alliance inventory scale scores. *Educational and Psychological Measurement*.

[17] Efron B, Tibshirani R (1993). *An Introduction to the Bootstrap*.

[18] Zhang L, Zhang B (2005). A quotient space approximation model of multiresolution signal analysis. *Journal of Computer Science and Technology*.

[19] Zhang L, Zhang B (2003). Theory of fuzzy quotient space (methods of fuzzy granular computing). *Journal of Software*.

[20] Han J, Kamber M (2001). *Data Mining: Concepts and Techniques*.

